# An In Vitro Study of the Effect of Cytotoxic Triorganotin Dimethylaminophenylazobenzoate Complexes on Red Blood Cells

**DOI:** 10.1007/s00232-018-0051-x

**Published:** 2018-10-22

**Authors:** Hanna Pruchnik, Aleksandra Włoch, Dorota Bonarska-Kujawa, Halina Kleszczyńska

**Affiliations:** Department of Physics and Biophysics, Wrocław University of Environmental and Life Sciences, ul. C.K. Norwida 25, 50-375 Wrocław, Poland

**Keywords:** Triorganotin compounds, Hemolytic activity, Erythrocyte, Cell membrane, Microscopy, Spectroscopy

## Abstract

Interactions of tributyltin (TBTA) and triphenyltin (TPhTA) 2-[4 (dimethylamino)phenylazo]benzoates, showing promising cytostatic activity against tumor cells, with erythrocytes and with erythrocyte membranes and model lipid membranes have been investigated. The effect of TBTA and TPhTA on the erythrocyte and its model membrane was investigated by the microscopic and spectroscopic methods. Interaction of tin complexes with the membrane was determined on the basis of hemolytic activity, changes induced in the shape of erythrocytes, as well as physicochemical parameters of the membrane, such as fluidity. The studies showed that the compounds in higher concentration induce hemolysis; however, TBTA is more toxic than TPhTA. Both TBTA and TPhTA induce morphological alterations in red blood cells—from discocytes to spherocytes and from discocytes to echinocytes. The results suggest that investigated complexes interact with the erythrocyte membrane, change its properties, and probably locate themselves in the hydrophilic part of the membrane, which agrees with conclusions drawn from investigation of erythrocyte membranes and model lipid membranes with the help of fluorescence and infrared spectroscopy.

## Introduction

**Fig. 1:**
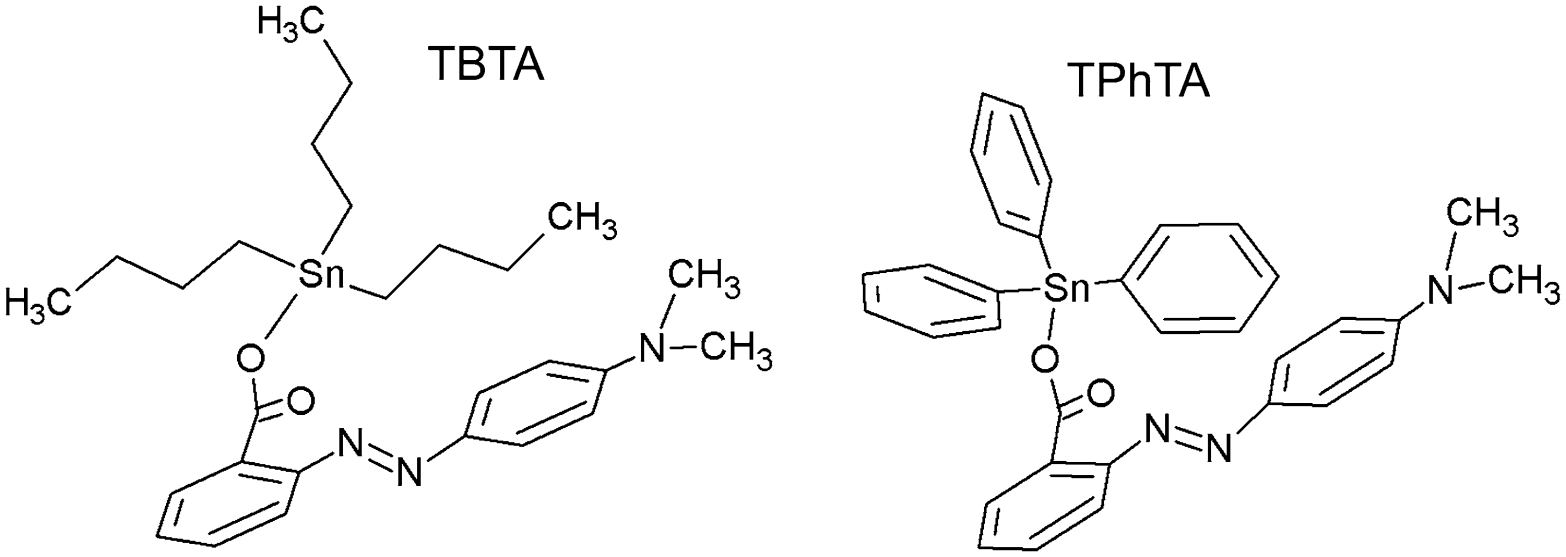
Chemical structure of TBTA and TPhTA

Metal complexes play essential role in agriculture and in pharmaceutical industry. The biological activity of organotin compounds has become well known due to their practical application as fungicides, bactericides, biocides, and pesticides. Recently, it was found that organotin compounds are very important in cancer therapy as potential anticancer agents. Some organotin(IV) complexes were found to have much higher activity in vitro than the currently used anticancer drugs; in particular, the highest activity was ascribed to tributyltin(IV) and triphenyltin(IV) compounds (mostly in nM concentration) (Gielen and Tiekink 2005; Pellerito and Nagy 2002; Nath et al. 2013; Williams et al. 2016; Baile et al. 2015; Edeler et al. 2017; Pruchnik et al. 2013, 2015, 2016). However, many of them show severe toxicity, which prompted a search for new compounds with high activity against tumor cells and decreased side effects (Hadjikakou and Hadjiliadis 2009; de Carvalho Oliveira and Santelli 2010; Ndagi et al. 2017; Basu Baul et al. 2017). The subjects of the research are tributyltin (TBTA) and triphenyltin (TPhTA) 2-[4 (dimethylamino)phenylazo]benzoate complexes (Fig. 1). These compounds (in short TTA) belong to the group of a very efficient anticancer agent. Previous studies in vitro showed their cytotoxic activity against human cell line A549 (lung adenocarcinoma) and HCV29T (bladder cancer cells). Both TBTA and TPhTA are very active against A549 cells (ID_50_ = 1.8–2.0 µmol dcm^−3^), also TPhTA (ID_50_ = 0.53 µmol dcm^−3^) turned out to be more active than cisplatinum (ID_50_ = 2.4 µmol dcm^−3^) in relation to human cell line HCV29T (Pruchnik et al. 2002, 2003). However, the mechanism of biological activity of the TTA is still unknown (Pruchnik et al. 2018). The action mechanisms of organotin derivatives on biological targets are often of diverse character or even not well known (Pettinari and Marchetti 2008). The cytotoxicity of organotin compounds may be caused by their interaction with lipid components of the membrane that may change its structure and function. Biological membranes are functional platforms for proteins and are susceptible to modifications induced by drug–lipid interactions that might modulate the location or activity of the membrane proteins. Drugs can have different effects on the membrane such as modification of the lipid conformation, surface charge, packing and lipid domains, fluidity of the membrane, its curvature, and, as a consequence—modification of cell functions. It is crucial to understand the interactions between potential chemotherapeutic agents and biological membranes, because it is directly related to the therapeutical activity and the toxicity of drugs (Alves et al. 2016; Schmidt et al. 2013; Lang et al. 2013). The red blood cells (RBCs) appear to be an excellent model to evaluate toxicity of molecules, natural or synthetic, by cellular damage measurement (Pagano and Faggio 2015; Faggio et al. 2015; Silva-Herdade et al. 2016). The hemolytic activity of a compound is an indicator of its general cytotoxicity towards normal cells (Arnold et al. 2013; Mischitelli et al. 2016a, b, c; Signoretto et al. 2016). RBCs are a good research model, because they do not have internal organelles, which make them an ideal cell system for studying basic drug–membrane interaction. Therefore, erythrocytes perform the basic functions assigned to the cell membrane (i.e., active and passive transport, formation of ion, and electric gradients), and on the other hand they have a simplified structure compared to other cell membranes (Bonarska-Kujawa et al. 2012; Włoch et al. 2015).

In present study, firstly, we have examined the hemolytic activity of the triorganotin dimethylaminophenylazobenzoates (TTA); secondly, we have examined the effect of TTA on red blood cells, such as changes induced in the shape of erythrocytes or in physicochemical parameters of the membrane, such as fluidity. To have a better understanding of the mechanism of the interaction of the complexes TTA with biological membranes, we used erythrocytes and molecular models of their membranes. The molecular models of red blood cell membranes consisted of lipids extracted from erythrocyte membranes. The effect of TTA on erythrocytes was examined by spectroscopic and microscopic methods, and the effect of TTA on erythrocyte membranes was investigated by fluorescence and infrared spectroscopy.

## Materials and Methods

### Materials

The complexes [Sn(C_6_H_5_)_3_{OOC-2-C_6_H_4_N=NC_6_H_4_N(CH_3_)_2_-4}]–TBTA and [Sn(C_4_H_9_-n)_3_{OOCC_6_H_4_N=NC_6_H_4_N(CH_3_)_2_-4}]–TPhTA (Fig. 1) were prepared by literature procedures described earlier (Pruchnik et al. 2002, 2003). The ^1^H, ^13^C, and ^119^Sn NMR and IR spectra were very similar to those given in the literature.

The studies were conducted on erythrocytes (RBCs) and on isolated erythrocyte membranes (RBCM), which were obtained from fresh pig blood using the Dodge method (Dodge et al. 1963). The pig blood used in our studies was acquired from Department of Internal Medicine and Clinic of Diseases of Horses, Dogs and Cats; The Faculty of Veterinary Medicine, University of Environmental and Life Sciences, Wrocław, Poland.

The choice of pig erythrocytes was dictated by the fact that the lipid composition of the membrane is very similar to that of the human erythrocyte, and the blood was readily available. Fresh blood was suspended each time in physiological solution of sodium chloride to which heparin had been added. Red blood cell lipids were extracted from erythrocyte membranes according to the method described by Maddy et al. (1972).

The fluorescent probes 6-dodecanoyl-2-dimethylaminonaphthalene (Laurdan), 1,6-diphenyl-1,3,5-hexatriene (DPH) were purchased from Molecular Probes and Merocyanine 540 (MC540) was purchased from Sigma Aldrich, Steinheim, Germany.

### Methods

#### Hemolysis

The hemolytic method used in our experiments determines the toxicity of the compounds and is based on that described by Łuczyński et al. (2013). Full blood was centrifuged (2500 rpm, 3 min) to remove the plasma and leucocytes, and next RBCs were washed three times with cold phosphate-buffered saline isotonic solution. The test sample (1 ml) contained an appropriate volume of phosphate-buffered solution, the compounds, and erythrocytes at a final hematocrit of 2%. The hemolytic activity of the compounds was determined for concentrations from 1 to 100 µM. Studied compounds were dissolved in ethanol in amounts such that after addition to erythrocyte suspension the concentration of ethanol in the samples did not exceed 1%. The control samples contained the same amounts of ethanol as the samples tested (but did not contain a compound).

Samples thus prepared were incubated for 1 h at 37 °C. After that 2 ml of phosphate buffer of pH 7.4 was added and the samples centrifuged (2500 rpm, 15 min) at room temperature. The extent of compound-caused hemolysis was determined on the basis of hemoglobin concentration in the supernatants. The measurement was performed at 540 nm wavelength using a UV–Vis spectrophotometer (Specord 40, Analytik Jena, Jena, Germany). Samples with total hemolysis (100%) were prepared by adding deionized water to the control samples. The percent of hemolysis was calculated using the following formula:

$$H\left( \% \right)=\frac{{{A_{\text{s}}}}}{{{A_{100\% }}}} \times 100\%,$$

where *H*(%) is the percentage of erythrocyte hemolysis, *A*_s_ is absorbance of hemoglobin in supernatant at 540 nm, and *A*_100%_ is the absorbance of hemoglobin in supernatant after complete hemolysis of the erythrocytes (100%).

### Microscopic Investigation

This method determines the location of compounds in the membrane based on the shape of blood cells, described earlier by Włoch et al. (2013). The optical instrument (Nikon Eclipse E200) equipped with a digital camera and scanning electron microscope (EVO LS15 ZEISS) were used in the experiments. For the investigation, RBCs were isolated from full blood as described in the hemolytic method. The test sample contained compounds (at final concentration 8 µM) suspended in 900 µL saline solution (0.9% NaCl) and 100 µL erythrocytes. The final concentration of erythrocytes in probes was 2% hematocrit. After that, the samples were incubated for 1 h at 37 °C. Next, the samples (after modification) were fixed with 2.5% solution of glutaraldehyde and after 30 min observed under microscope to count erythrocytes of various shapes.

Before observation with a scanning electron microscope, the samples after incubation were fixed with 2.5% glutaraldehyde for 48 h and washed in a phosphate buffer for 20 min. Next, to dehydrate the samples, they were washed in acetone solution of various concentrations (30, 50, 60, 70, 80, 90%) for 15 min. In 100% concentration of the acetone solution, the samples were left for 30 min, and then dried for 12 h at room temperature. After that, the samples were subjected to microanalysis with a Brucker AXS Quantax Roentgen microanalyzer. At the end, the samples were coated with gold and analyzed using a scanning electron microscope.

Different shapes of erythrocytes were classified according to the Bessis and Brecher scale (Deuticke 2003). In this scale, appropriate morphological indexes are assigned to three basic shapes: (0) discocytes, (−) stomatocytes, and (+) echinocytes.

### Fluorescence Spectroscopy

The effect of organometallic tin on fluidity and packing arrangements of lipids in the red blood cell membrane (RBCM) and a model lipid membrane (RBCL) was investigated using fluorimetric method described earlier by Włoch et al. (2015). Small unilamellar liposomes were used as a model lipid membrane. The liposomes were formed from natural lipids extracted from erythrocytes (RBCL) and the procedure was described in detail by Bonarska-Kujawa et al. (2014). First, a mixture of lipid membranes with fluorescent probes was prepared. The mixture contained RBCM or RBCL suspended in isotonic phosphate solution of pH 7.4 and fluorescent probes at a concentration of 1 µM and were incubated for 30 min in darkness at room temperature. After that, studied compounds at 8–80 µM were added to the mixture and incubated for 1 h at 37 °C. After 1 h, measurements were conducted with a CARY Eclipse of VARIAN fluorimeter at 37 °C. Control samples contained only a mixture with addition of ethanol.

In our experiments, we used fluorescent probes such as Laurdan, MC540, and DPH located in different areas of membrane. DPH provides information about the hydrophobic regions of phospholipid bilayers, and Laurdan about hydrophilic regions (at the level of the phospholipid glycerol backbone). Merocyanine 540 was used as a probe to monitor the molecular packing of phospholipids in the outer leaflet of membrane. The MC540 probe is a heterocyclic chromophore with a localized negative charge that binds to the outer leaflet of the phospholipid bilayer of membranes. Binding of MC540 to phospholipid bilayers depends on the packing of the bilayer of a membrane (Lagerberg et al. 1997).

The excitation and emission wavelengths for DPH and MC540 probes were *λ*_ex_ = 360 nm, *λ*_em_ = 425 nm, and *λ*_ex_ = 550 nm, *λ*_em_ = 585 nm, respectively. The excitation wavelength for Laurdan was 360 nm, and the emitted fluorescence was recorded at two wavelengths: 440 and 490 nm.

Changes in fluidity of the hydrophobic regions of the phospholipid bilayers were detearmined on the basis of changes in fluorescence anisotropy (A) for DPH probe, and A was calculated using the following formula (Lakowicz 2006):

$$A=\frac{{({I_{{\text{II}}}} - G{I_ \bot })}}{{({I_{{\text{II}}}}+2G{I_ \bot })}},$$

where *I*_II_ and *I*_⊥_ are the fluorescence intensities observed in directions parallel and perpendicular, respectively, to the polarization direction of the exciting wave. *G* is an apparatus constant dependent on the emission wavelength. Changes in the polar group packing arrangement of the hydrophilic part of the membrane were investigated using Laurdan probe, on the basis of generalized polarization (GP) and were calculated with the formula (Lakowicz 2006):

$$GP=\frac{{({I_{\text{b}}} - {I_{\text{r}}})}}{{({I_{\text{b}}}+{I_{\text{r}}})}},$$

where *I*_b_ is the fluorescence intensity at $\lambda$_em_ = 440 nm and *I*_r_ is the fluorescence intensity at $\lambda$_em_ = 490 nm.

### FTIR Spectroscopy

The FTIR method was used to determine the molecular interactions between the compounds and specific functional groups of lipids. This method was described in details by Włoch et al. (2015). In the experiment, we used the red blood cell membranes (RBCM) as a model lipid membrane. The studied samples containing the RBCM (suspended in physiological salt) and organometallic tin compounds at 4 µM concentration were incubated for 24 h at 37 °C. The control samples contained RBCM and added ethanol. Next, the samples were centrifuged (30000*g* × 15 min) and 50 µL of condensed RBCM was applied at the ZnSn plate. Then, to remove the water, the plate was situated for 24 h in incubator. After incubation, the measurements were performed using a Thermo Nicolet 6700 MCT (Thermo Fisher Scientific, Waltham, MA). Bands from vibrations of CH_2_ and CH_3_ groups of alkyl chains, the phosphate group (PO_2_^−^), and the trimethyl ammonium group were examined.

### Statistical Analyses

Statistical analysis was carried out using Statistica 9.0 (StatSoft Inc.). Measurements, using various methods, were carried out in triplicate, with specified exceptions. Using Dunnett’s post hoc test, the analysis of variance was conducted as well as significance between means was determined. Results were presented as mean ± SD. Significance levels were defined at *p* < 0.05.

## Results and Discussion

### Hemolytic Activity

**Fig. 2:**
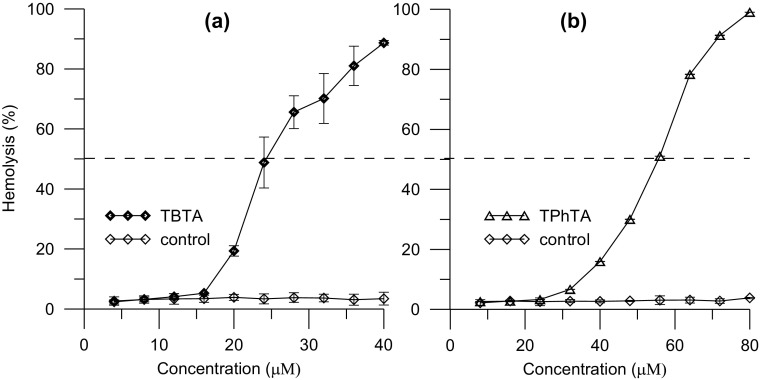
The dependence of percentage of erythrocyte hemolysis on the concentration of tributyltin and triphenyltin complexes

To examine the hemolytic activity of the triorganotin dimethylaminophenylazobenzoate complexes (TTA), the spectrophotometric method was used. To measure the toxicity of TTA compounds, the extent of lysis was assayed. The experiment was carried out in a wide range of TTA concentrations, from 1 µM up to 100 µM. After incubating the RBCs in the presence of investigates complexes, it was observed that the lysis of erythrocytes increased. The suspension of unmodified erythrocyte cells was used as a control probe. The extent of hemolysis was estimated from the amount of the extracellular hemoglobin. The relationship between percentage of hemolysis and the concentration of investigated compounds is presented in Fig. 2. On the grounds of the obtained results, it may be stated that both TBTA and TPhTA induce hemolysis; however, the TBTA (Fig. 2a) complex shows higher activity than TPhTA (Fig. 2b). The 50% hemolysis (C_50_) was observed at 25 µM concentration for TBTA and 57 µM for TPhTA. The higher toxicity of TBTA in comparison with TPhTA most probably should be attributed to differences in their structure. Due to that, the hydrophobic chains of TBTA are able to penetrate the lipid bilayer deeper than the phenyl rings of TPhTA. It is well known that toxicity of organotin compounds depends not only on the number of organic groups attached to the tin but also on the nature of the organic group (Pettinari and Marchetti 2008; Pruchnik et al. 2013, 2015).

### Microscopic Investigation of Erythrocytes

**Fig. 3:**
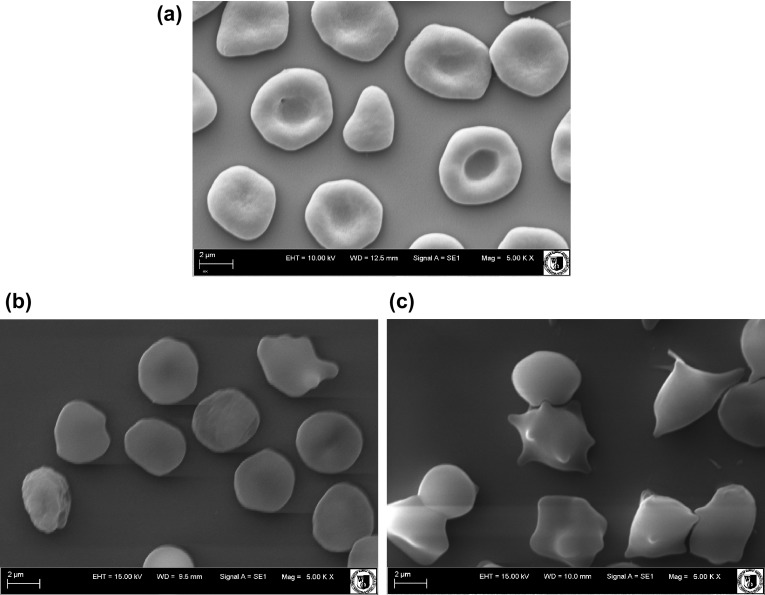
Photographs of red blood cells modified with TTA complexes at 8 µM, obtained with an electron microscope: control (**a**), TBTA (**b**), TPhTA (**c**)

The examination of shapes of erythrocytes using optical and electron microscopes shows that the TTA complexes induce morphological changes in RBCs. For normal RBCs, the biconcave disc (discocyte) is a typical shape (Fig. 3a). It is possible to transform the normal cell into other shapes by exposing it to different external conditions. The changes in the geometric and biophysical characteristic of the erythrocyte, like the surface area-to-volume ratio, the viscosity of the cytoplasm, and the elasticity of the membrane, are good characteristics of the deformation of the cell (Muñoz et al. 2010). RBCs shapes were classified according to Bessis and Brecher’s scale (Deuticke, 2003) where various shapes are given following morphological indices: spherostomatocytes (− 4), stomatocytes II (− 3), stomatocytes I (− 2), discostomatocytes (− 1), discocytes (0), discoechinocytes (1), echinocytes (2), spheroechinocytes (3), spherocytes (4) (Table 1).

**Table 1:** Individual erythrocyte shapes formed in the presence of organotin compounds at a concentration of 8 µM (% ± SD)

Shape of erythrocytes	Control	TPhTA	TBTA
Spherostomatocytes (− 4)	0.00	0.00	0.00
Stomatocytes II (− 3)	0.00	0.00	0.00
Stomatocytes I (− 2)	1.00 ± 0.67	0.08 ± 0.00	0.25 ± 0.00
Discostomatocytes (− 1)	3.66 ± 0.79	1.69 ± 0.32	0.12 ± 0.00
Discocytes (0)	62.33 ± 2.63	22.88 ± 1.40	5.40 ± 3.73
Discoechinocytes (1)	24.60 ± 1.67	26.27 ± 1.67	0.00
Echinocytes (2)	8.37 ± 1.29	40.69 ± 1.63	0.00
Spheroechinocytes (3)	0.00	7.66 ± 1.26	0.82 ± 0.55
Spherocytes (4)	0.00	0.73 ± 4.95	93.44 ± 4.18

Photographs obtained from an electron microscope (Fig. 3b, c) indicate that investigated compounds change cell shapes mainly into echinocytes. The TTA complexes induce various forms of echinocytes, percent share of which depending on the type of the compound. The share of various forms of cells in a population of erythrocytes modified with the compounds at 8 µM concentration is shown in Table 1. In the presence of TBTA, more than 90% of cells took the shape of spherocytes (4), whereas TPhTA changed the shape of cells mainly to discoechinocytes (1) and echinocytes (2).

According to the Sheetz and Singer hypothesis, echinocytes are created when the modifying compound is concentrated mainly in the outer lipid layer, while stomatocytes are formed when the substance is accumulated in the inner lipid layer of the RBCs membrane (Sheetz and Singer 1974; Iglic et al. 1998; Lim et al. 2002). It was also observed that when the concentration of echinocytogenic molecules is further increased, spherocytes began to appear and finally the process of hemolysis begins (Brecher and Bessis 1972; Isomaa et al. 1987; Hägerstrand and Isomaa 1989, 1992; Iglic et al. 1998; Suwalsky et al. 2009, 2016, 2017, 2018; Bonarska-Kujawa et al. 2012, 2014). In accordance with the hypothesis presented above, investigated complexes are expected to be localized mainly in the outer lipid monolayer of the erythrocyte membrane.

As noted in the previous section, the hemolytic analysis indicated that TBTA is more toxic than TPhTA. This conclusion is supported by the results of morphological analysis—at the same concentrations, the compound TBTA deformed erythrocytes mainly into spherocytes, whereas for TPhTA no spherocytes were observed at all. Probably, only at much higher concentrations TPhTA would cause spherocytes to form. But further increase in the concentration of the compounds leads to rupture of the cell membrane and hemolysis of the erythrocytes.

### Fluorescence and FTIR Spectroscopy of Erythrocyte Membrane (RBCM) and Natural Lipids Small Unilamellar Vesicles (RBCL)

Fluorescence spectra are source of information referring to hydration, rigidity, and packing order of molecules that are used to build the membrane system. To determine the impact of the TBTA and TPhTA complexes on physicochemical properties of the erythrocyte and lipid model membranes, the selected fluorescence probes (Laurdan, MC540, and DPH) were used. The selection of these probes was justified by the fact that they allow to obtain information about the physical state of phospholipids in different regions of the bilayer.

**Fig. 4:**
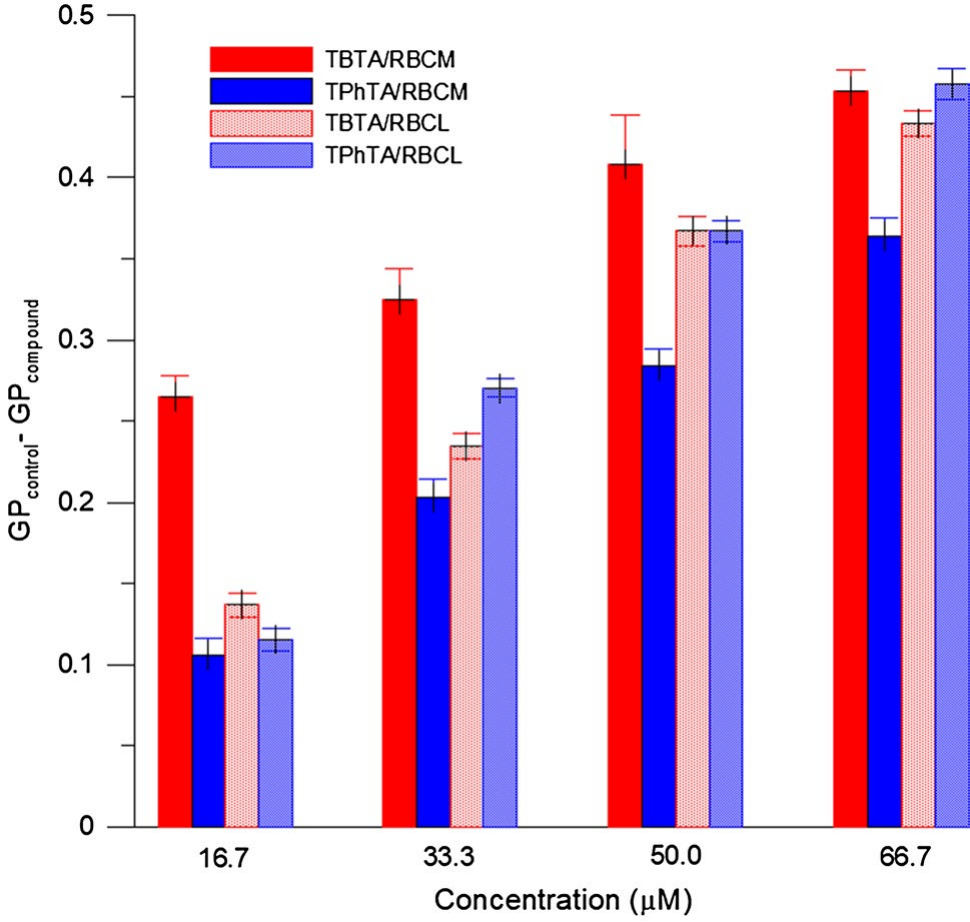
Differences in values of generalized polarization (GP) of the Laurdan probe for RBCM and RBCL modified with triorganotin complexes at 37 °C

Laurdan probe is sensitive to polarity changes and dynamic properties at the membrane lipid–water interface. In the lipid membrane, the location of the maximum Laurdan emission strongly depends on the degree of hydration of the bilayer. In the gel phase when lipids are hydrated to a small extent and solvent relaxation does not occur, the maximum emission of Laurdan is about 440 nm. In the liquid crystalline phase, the bilayer relaxes, which eases penetration of water molecules into the region of the fluorescence probe. Then the energy of the excited state is transferred to the water dipoles which start re-orienting around the fluorophore causing its emission band to shift towards longer wavelengths (Parasassi et al. 1998; Sanchez et al. 2007). Polarity changes are shown by shifts in the Laurdan emission spectrum, which are quantified by calculating the generalized polarization (GP). The calculated values of GP, both for RBCL and for RBCM, are significantly decreased with increasing concentration of investigated complexes—both TBTA and TPhTA. Figure 4 shows the difference between GP value for pure membrane and membrane modified with the investigated complex of specified concentration (GP_control_ − GP_compound_). It is evident that in the presence of TTA complexes (and TBTA in particular) the changes of GP are more pronounced for RBCM than for RBCL. Moreover, for RBCM the differences between how TBTA and TPhTA influences the GP become more apparent.

The decrease in GP values in the presence of TTA complexes suggests that both TBTA and TPhTA affected lipid head group packing and hydration in such a way that more water molecules were incorporated at the hydrophobic–hydrophilic interface of the model membrane. In comparison with TPhTA, TBTA causes slightly higher increase of disorder in the hydrophilic part of the lipid layer for RBCM. Changes induced by investigated compounds in RBCL liposome membranes are smaller than those in erythrocyte membranes (Fig. 4). This suggests that the compounds are incorporated into erythrocyte and liposome membranes, concentrating mainly in their hydrophilic parts. Changes induced by TBTA are slightly more pronounced which would suggest increased number of water molecules present at the interface of the bilayer and decreasing order of the polar heads of the lipid bilayer.

**Fig. 5:**
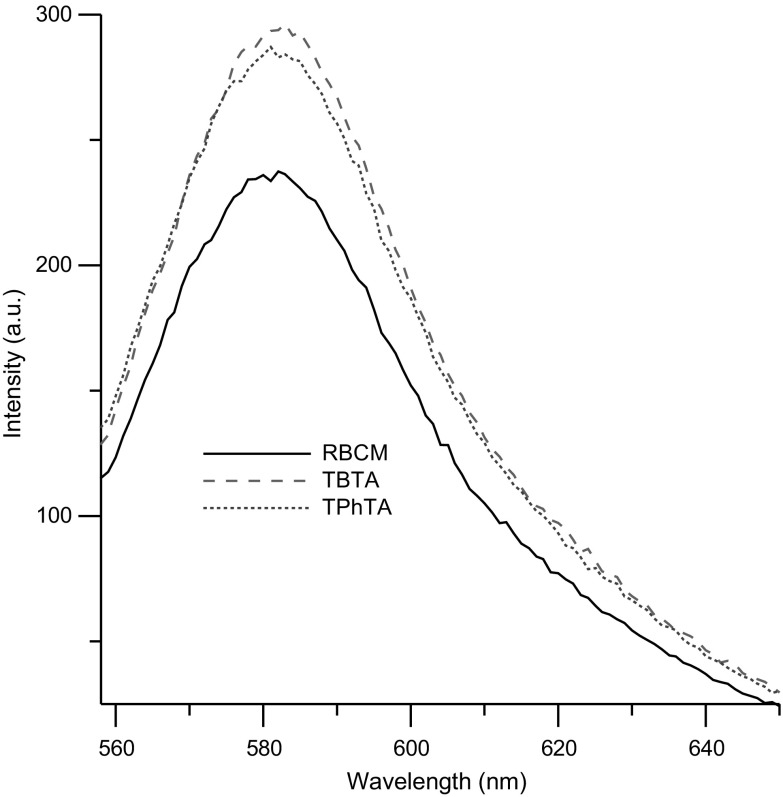
Fluorescence emission spectra of MC540 in pure RBCM (solid line) and modified with 8 µM TTA (dashed lines)

Experiments performed with MC540 probe lead to similar conclusions. The MC540, similarly to Laurdan, is sensitive to polarity of the environment, and the negative charge of the amphiphilic anion molecule determines its location at or near the membrane interface, slightly above the glycerol backbone of lipids. It has been shown that fluorescence intensity of MC540 strongly increases in the liquid phase of lipid bilayers compared to lipid in the gel phase; therefore, this probe is very sensitive to the lipid packing of the membrane (Alay et al. 2003; Langner and Hui 1999; Manrique-Moreno et al. 2014). In our study, the enhanced fluorescence intensity of MC540 was observed both for TBTA and TPhTA (Fig. 5). This result suggests that organization of the lipids decreased, which would indicate that membrane surface area accessible for the binding of the dye increased due to the loss in lipid packing order (Langner and Hui 1999; Manrique-Moreno et al. 2014).

The effect of TTA complexes on fluidity of the RBCM and RBCL was studied with the help of anisotropy measured with the DPH probe. DPH reflects the perturbation in the hydrophobic part of the lipid bilayer. The fluorescence steady-state anisotropy is primarily related to the restriction of the rotational motion of the dye, and thus to the hydrocarbon chain packing order. Therefore, a decrease of the anisotropy parameter can be explained by a structural perturbation in the bilayer’s hydrophobic part, probably due to incorporation of studied compounds. The dependence of the fluorescence anisotropy of DPH probe on investigated compounds is presented in Table 2. The presence of TTA practically does not change fluorescence anisotropy for RBCL. For higher concentrations of TBTA, we observed only slight increase in the anisotropy. More pronounced changes were observed for RBCM, in particular when it is modified with TBTA the anisotropy increased. These results suggest that TPhTA has practically no influence on fluidity in the hydrophobic region of the lipid bilayer. Slight increase in anisotropy for TBTA may indicate that this compound influences packing order of hydrophobic chains. On the other hand, a significant drop of GP suggests increased number of water molecules in the interphase area (Table 2).

**Table 2:** Values of fluorescence anisotropy of DPH probe for RBCM and RBCL modified with different concentrations of TTA at 37 °C

Concentrations (µM)	Anisotropy (A) ± SD			
	RBCM		RBCL	
	TBTA	TPhTA	TBTA	TPhTA
0	0.229 ± 0.001	0.229 ± 0.001	0.216 ± 0.003	0.216 ± 0.003
16.7	0.236 ± 0.001	0.231 ± 0.001	0.219 ± 0.002	0.215 ± 0.001
33.3	0.248 ± 0.001	0.230 ± 0.002	0.224 ± 0.002	0.214 ± 0.002
50.0	0.257 ± 0.006	0.233 ± 0.002	0.227 ± 0.003	0.216 ± 0.004
66.7	0.266 ± 0.007	0.236 ± 0.003	0.229 ± 0.008	0.219 ± 0.004

An increase in head group size leads to increased chain tilting and creates energetically unfavorable voids in the hydrocarbon region of non-interdigitated membranes, encouraging the creation of the non-lamellar phase at high concentrations, which would explain the slight increase in anisotropy (Nagel et al. 1992; Boggs and Tummmler 1993; Epand 1996; Ahl and Perkins 2003). Our results suggest that toxicity of TTA compounds may be related to structural changes they induce in the bilayers. This agrees with the suggestion that interdigitated gel phase as well as the inverted hexagonal gel phase can play an important role in regulating many functions of biomembranes (Komatsu and Okada 1995a, b).

**Fig. 6:**
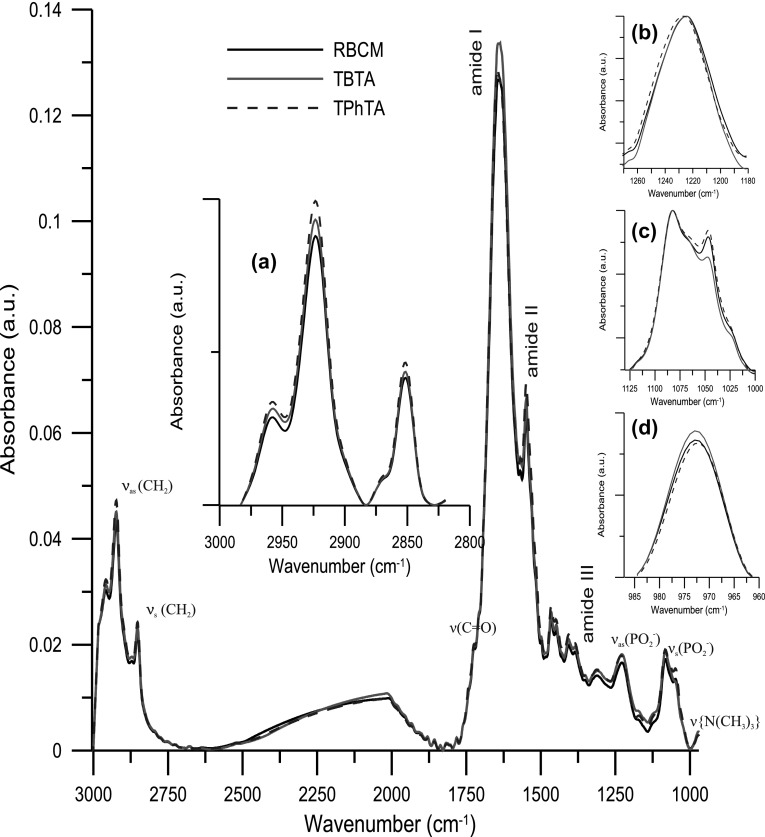
FTIR spectra of RBCM pure (solid line) or containing incorporated organometallic compounds (thin line): **a** symmetric and asymmetric CH_2_ stretching band, **b** phosphate band I (asymmetric vibration), **c** phosphate band II (symmetric vibration), **d** choline band

Additionally, to get more detailed information about molecular mechanisms of interaction of TBTA and TPhTA with erythrocytes membranes, the FTIR method was employed. The structural changes in RBCM were observed simultaneously at the level of hydrophilic groups of lipid, in a hydrophobic part composed of hydrocarbon lipid chains as well as in the lipid membrane interface represented by ester lipid groups. Figure 6 shows comparison of infrared spectra of RBCM and RBCM doped with TPhTA and TBTA. The most intense vibration in lipid systems is the CH_2_ stretching vibration and these give rise to bands in the 3000–2800 cm^−1^ region. The increase in the wavenumber of these bands testifies to an increase in fluidity of the hydrophobic part of the membrane. Both TBTA and TPhTA do not change the frequency of signal from hydrocarbon chains. For this reason, we can assume that the tested compounds do not significantly affect the hydrophobic regions of the lipid bilayer (Fig. 6a). The interaction of TTA and the head group of RBCM was monitored by analyzing the symmetric and asymmetric phosphate bands as well as choline stretching bands (Fig. 6b–d). The frequency range of 1220–1260 cm^−1^ corresponds to the asymmetric stretching vibration of PO_2_^−^ phosphate groups (Fig. 6b). The *ν*_as(PO2_^−^_)_ frequency band exhibits high sensitivity to changes of the environment polarity and to possibility of interaction via hydrogen bonds. During the lipid bilayer hydration process, the number of phosphate groups interacting with water molecules increases and as a result the maximum of the band moves towards lower wavenumbers. In the presence of TTA, the frequency of oscillation is slightly shifted to higher values (for pure RBCM the *ν*_as(PO2_^−^_)_ = 1224.49), *ν*_as(PO2_^−^_)_ = 1224.79, and *ν*_as(PO2_^−^_)_ = 1226.71 for TBTA and for TPhTA, respectively. Perhaps, TTA compounds form a bond between oxygen atom of the phosphate group of the phospholipid and the tin atom and hence the change of hydration of the phosphate group. For symmetric vibration, the values of wavenumbers changes very slightly (*ν*_s(PO2_^−^_)_ = 1082.47, *ν*_s(PO2_^−^_)_ = 1082.18 (TBTA) and *ν*_s(PO2_^−^_)_ = 1082.36 (TPhTA), Fig. 6c). Position of the maximum of the band derived from *ν*_s_(PO^2−^) vibration primarily reflects changes in the conformation of the phosphate part of the lipid. The changes of values of wavenumbers in the presence of TTA indicate slight changes in the conformation of the polar lipid groups. Additionally, in the presence of TPhTA the band of the phosphate group is much broader than for pure RBCM, indicating that there is an interaction between the investigated compound and the lipid. The band observed in the polar part of the lipid spectra corresponds to the vibration in the choline fragment with the maximum at *ν*_as(N−C)ip_ = 971.9 for pure RBCM. Analysis revealed that TTA induce very slight changes in this band and only in liquid phase (*ν*_as(N−C)ip_ = 971.9 for TBTA and *ν*_as(N−C)ip_ = 971.76 for TPhTA). Unfortunately, because of the complexity of the system (hydrated protein–lipid membrane), the range of ester groups (1750–1700 cm^−1^) is not clearly visible because protein bands overlapped with water molecule bands. In the range of 1610–1695 cm^−1^, Amid I band exists with dominating ν_(C=O)_ contribution. Therefore, it is not possible to explicitly confirm conclusions drawn from fluorimetric measurements about increased hydration of the ester group region. The interesting thing is that in the presence of investigated compounds a slight shift of the maximum of the Amid I band is observed, which may implicate that proteins present in the membrane underwent some structural changes.

**Fig. 7:**
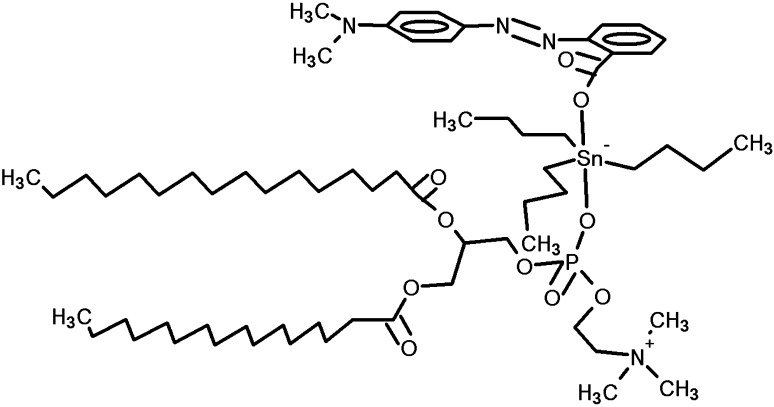
Probable bond between oxygen atom of phosphate group of phospholipid and tin atom of SnBu_3_(OOCR) complex

Taking everything into account, data presented in the paper show that TBTA and TPhTA interact with erythrocyte membranes, locating themselves in the lipid head group region and altering properties of the membrane. These complexes, by slightly influencing the fluidity of the lipid bilayer (especially TBTA), modify the packing of lipid heads and probably change the hydration of the membrane. Our studies showed structure–activity relationship of investigated compounds: TBTA is more hemolytically active/toxic than TPhTA. The tin atoms in TBTA complex, in the solid state, are five-coordinate with axial coordination sites occupied by oxygen atoms bridging carboxylate ligand. In solutions, polymeric TBTA complex dissociate, giving also trigonal bipyramidal SnBu_3_(OOCR)(solv) complexes with RCOO and solvent molecule coordinated in axial positions (Pruchnik et al. 2002, 2003, 2018). In the presence of water in solutions, H_2_O is most probably coordinated to the Sn atom. The TPhTA in solid state and in solutions has pseudotetrahedral structure, because phenyl groups are more sterically demanding ligands than butyl groups. The TBTA can probably form a bond between oxygen atom of the phosphate group of phospholipid and the tin atom (Fig. 7). However, formation of strong hydrogen bonds between aqua ligand of [SnBu_3_(OOCR)(H_2_O)] complex and oxygen atoms of phospholipids is also possible (Pruchnik et al. 2018). Effective interaction of polar head groups with TBTA leads to considerable changes of the hydrophilic layer of the membrane, which are bigger than those caused by TPhTA. This causes synergistic increase of the water content in the polar part of the membrane and therefore enhanced structural changes. This effect is smaller in the case of TPhTA.

## Conclusion

In order to explain the molecular mechanism of the toxicity of tributyltin and triphenyltin complexes with 2-[4-(dimethylamino)phenylazo]benzoate (TTA), the interaction of these compounds with erythrocytes and with lipid model membrane of erythrocytes was analyzed.

Our results, obtained using optical and electron microscopy, spectroscopy, and fluorimetry methods, unambiguously showed that TTA compounds interacted with the erythrocyte membrane and altered its properties. Both TBTA and TPhTA in higher concentrations induce hemolysis, though not to the same extent. TBTA is about twice as hemolytically active/toxic as TPhTA. This is probably caused by a slightly different structure of the compounds. The analysis of erythrocyte shapes showed that these complexes induce morphological changes in erythrocytes. The normal discoid shapes are transformed mainly to the echinocytic form. Thus, it may be concluded that TTA complexes are localized mainly in the outer monolayer of the erythrocyte membrane, though the hydrophobic chains of TBTA are able to penetrate the lipid bilayer deeper compared to the phenyl rings of TPhTA. Measurements using fluorescence probes showed that tested compounds altered interfacial membrane hydration, changed packing order of lipid polar heads, and caused a slight reduction of the fluidity of the hydrophobic part of the lipid bilayer. FTIR measurements showed a change mainly in the polar region of the lipid bilayer—most probably TTA forms a bond between the oxygen atom of the phosphate group of the phospholipid and the tin atom.

Summarizing, our results suggest that toxicity of TTA compounds may be related to their effect on membrane properties, e.g., hydration and fluidity, and/or to the structural changes they induce in the membrane by binding to specific molecular targets.
